# Frequency of Thyroid Nodules among Patients with Colonic Polyps

**DOI:** 10.1155/2012/178570

**Published:** 2012-01-26

**Authors:** Cevdet Duran, Huseyin Savas Gokturk, Mustafa Kulaksizoglu, Suleyman Bakdik, Gulhan Kanat Unler, Mustafa Erbayrak, Guven Ozkaya, Baris Onder Pamuk, Mustafa Sait Gonen

**Affiliations:** ^1^Division of Endocrinology and Metabolism, Konya Training and Research Hospital, 42100 Konya, Turkey; ^2^Division of Gastroenterology, Konya Research Hospital, Baskent University, 42080 Konya, Turkey; ^3^Division of Endocrinology and Metabolism, Meram Medical School, Selcuk University, 42080 Konya, Turkey; ^4^Department of Radiology, Konya Training and Research Hospital, 42100 Konya, Turkey; ^5^Division of Gastroenterology, Yasam Hospital, 07960 Antalya, Turkey; ^6^Department of Biostatistic, Medical School, Uludag University, 16059 Bursa, Turkey; ^7^Division of Endocrinology and Metabolism, Bozyaka Training and Research Hospital, 35110 Izmir, Turkey

## Abstract

*Aim*. Colonic polyps and thyroid nodules are common diseases and their frequency increases with age. In the literature, there is no study investigating the coexistence of colonic polyps and thyroid nodules. Therefore, this study was designed to investigate thyroid nodule prevalence in patients with colonic polyps. *Material and Methods*. Sixty-six patients with colonic polyps and 146 patients without colonic polyps enrolled into the study. Age and sex matched control group was composed from patients without colonic polyps. Colonoscopic examinations, thyroid ultrasonographies were performed in all patients, and TSH were measured. *Results*. Male/female ratio in polyp and control groups were 40/26 versus 68/78, respectively (*P* = 0.058). Mean ages were similar in both groups (53.3 ± 11.4 versus, 51.8 ± 11.4, *P* = 0.373). Thyroid nodule was detected in 44 (66.7%) patients with polyps and in 61 (41.8%) controls (*P* = 0.001). Patients with adenomatous polyps had 5 or more thyroid nodules compared to patients with hyperplastic polyps (*P* = 0.03). Thyroid nodules were more prevalent among patients aged 50 or older compared to 50 years or less (*P* = 0.023). *Conclusion*. Thyroid nodules were detected more common in patients with colonic polyps. Further studies are needed to clarify this coexistence.

## 1. Introduction

Colonic polyps and thyroid nodules are very common diseases in the general population and their prevalence increases with age [1, 2]. Epidemiological, clinicopathological, and molecular genetic studies suggest that colorectal adenomas (about two-thirds of all colonic polyps) are now considered precursors of colonic adenocarcinomas. It has been shown that the screening and resection of the adenomas in asymptomatic population provides a powerful tool to reduce colorectal cancer incidence. Although the etiology of colorectal adenomas is not well understood, there are large numbers of clinical environmental and lifestyle factors that are associated with increased risk of colorectal adenomas [3].

Like colonic adenomas, thyroid nodule prevalence increases with age in certain populations like females or in iodine deficient regions. Ultrasound (US) and autopsy studies report that thyroid nodule prevalence ranges from 19% to 50% [4, 5]. Based on our clinical observations, thyroid nodules are more commonly seen in patients with colonic polyps. In the literature, there is no study investigating the coexistence between colonic polyps and thyroid nodules. Therefore, this study was designed to investigate thyroid nodule prevalence in patients with colonic polyps.

## 2. Material and Methods

This study is conducted in Divisions of Endocrinology and Gastroenterology of Konya Training and Research Hospital and Baskent University Medical Faculty Konya Training Hospital, between 2008-2009. All colonic investigations were evaluated, and patients with neoplastic colonic polyps were enrolled in the study. Patients with all types of malignancies, ulcerative colitis, submucosal, mucosal, inflammatory pseudopolyp, known familial polyposis coli syndrome, usage of l-thyroxine or antithyroid drug were excluded. After enrollment, thyroid US was performed and all nodules detected by US were recorded on patients form. Thyroid stimulating hormone (TSH) and free thyroxine levels were measured. Sixty-six patients with colonic polyps and age and sex-matched 146 patients without colonic polyps, as a control group, included in the study. Procedures were applied in agreement with ethical committee.

Colonoscopic examinations were performed by Olympus CF-Q180 AL video colonoscope, and the number and localization of the polyps were recorded. When polyp was detected, and biopsy and resection was performed, pathological characteristics were recorded. Thyroid US scanning was performed using the 5–13 MHz linear probe, by Siemens Acuson Antares. We considered as thyroid nodules all the US nodular lesions ≥3 mm. The number of thyroid nodules were divided according to the largest diameter as <10 mm and ≥10 mm and recorded.

All statistical analyses were performed with SPSS ver.17.0. Shapiro Wilk test was used as normality test. Continuous variables were compared with Student *t*-test for normally distributed data and Mann-Whitney *U* test for nonnormal distributed data. Categorical variables were compared using Pearson's chi-squared test and Fisher's exact test. A *P* value < 0.05 was considered as significant.

## 3. Results

**Figure 1:**
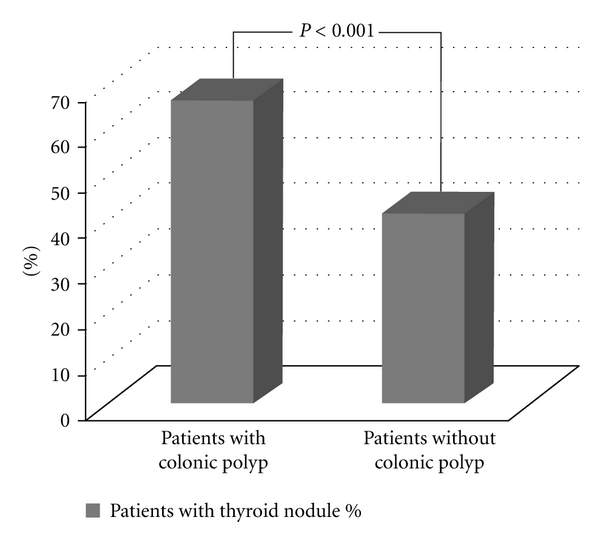
Percentage of patients with or without thyroid nodules.

In this period, 965 colonoscopies were performed and 191 patients with colonic polyps were detected. After the exclusion, patients were asked to participate, among them 66 with colonic polyp, and a total of 212 patients were included into the study. Male/female ratio in polyp and control groups were 40/26 versus 68/78, respectively (*P* = 0.058). Mean ages were similar in both groups (53.3 ± 11.4 versus, 51.8 ± 11.4, *P* = 0,373). Thyroid nodule prevalence was higher in patients with colonic polyps (44/66, 66.7%) than controls (61/146, 41.8%), (*P* = 0.001), (Figure 1). Forty-six patients had single polyp, 9 had 2 polyps, 7 had 3 and 4 had 4 or more polyps. Most of them were localized in rectosigmoidal region. 

Histopathological examination revealed adenomatous polyp in 36 (55%) patients, hyperplastic polyp in 20 (30%) patients, and mixed type in 10 (10%). None of the polyps were malignant. In the polyp group, 46 patients had single polyp and 31 of them had thyroid nodules, 20 patients had 2 or more polyps and among these, 13 patients had thyroid nodules (*P* = 0.85).

In patients with colonic polyp, 36 patients had 4 or less thyroid nodules and 8 patients had 5 or more thyroid nodules. Patients with adenomatous polyps had 5 or more thyroid nodules compared to patients with hyperplastic polyps (in the hyperplastic polyp group, there was not any patients with 5 or more thyroid nodules) (*P* = 0.033).

When patients with thyroid nodules were classified, 95 patients had 4 and less than 4 thyroid nodules and 10 patients had 5 or more thyroid nodules.

Mean TSH levels were lower in the polyp group than controls (1.17 ± 0.95 versus 1.54 ± 1.3, *P* = 0.002, resp.). Thyroid nodules were greater than 10 mm in 34 (32%) patients. Age has no implication on colonic polyp frequency; thyroid nodules were more prevalent among patients aged 50 or older compared to 50 years or less (under age 50; 34/85, 50 or older; 71/127) (*P* = 0.023).

Colonic polyp, or thyroid nodule existence were similar between sexes (*P* = 0.63 and *P* = 0.182, resp.).

## 4. Discussion

This is the first study investigating that concomitance between colonic polyp and thyroid nodules, and we found a higher thyroid nodule prevalence in patients with colonic polyps. Also we found lower TSH levels in patients with colonic polyps; patients with adenomatous polyps had 5 or more thyroid nodules compared to patients with hyperplastic polyps and thyroid nodules were more prevalent among patients aged 50 years or older.

Colonic polyp and thyroid nodules are common disorders and their prevalence increases with age. Ultrasound studies and autopsy studies report a prevalence of thyroid nodules up to 50% in elderly patients and in women [4, 5]. Thyroid nodule prevalence is higher in iodine-deficient area. In our country, iodine propyhylaxis was started in 1999 and our region is the mildly iodine-deficient area at the moment (average urinary iodine concentration 92 *μ*g/L) [6]. Similarly, colonic polyp prevalence increases up to 50% with age 70 [2]. Although both disorders have different etiopathogenic mechanism, coexistence of colonic polyp and thyroid nodules mechanism is unknown, yet.

Recently, there are some studies pointing out that insulin resistance may be the common mechanisms. Kurimoto et al. reported that colonic polyp prevalence is higher in patient with acromegaly or insulin resistance [7]. In both situations high insulin or insulin-like growth hormone-1 levels may cause increased polyp formation. In patients with metabolic syndrome, colonic polyp prevalence is increased and insulin resistance is responsible for this situation [8, 9].

Similarly, some studies reported a coexistence between insulin resistance and thyroid nodules. In 2008, Rezzonico et al. reported that thyroid volumes, measured by ultrasonography, were higher in patient with insulin resistance than patients without insulin resistance [10]. The same group reported that insulin resistance would be an important risk factor for developing differentiated thyroid carcinoma [11]. Rezzonico et al. reported that metformin treatment decreases thyroid nodule volume in patients with small thyroid nodule and insulin resistance [12].

Ayturk et al. reported that patients with metabolic syndrome have higher thyroid nodule prevalence and nodule volume and demonstrated that insulin resistance may be a major independent risk factor for development of nodule formation in iodine-deficient area [13]. To the best of our knowledge, there is no published study investigating coexistence of colonic polyp and thyroid nodule, so this study was not designed to evaluate common mechanism. Therefore, we did not evaluate insulin resistance and this is a major limitation of this study.

Although, there was no patient with thyrotoxicosis, another unexpected result of the study is lower TSH levels in patients with colonic polyp. This situation can be speculated as patients with colonic polyp have higher thyroid nodules prevalence and iodine prophylaxis may results autonomy in some nodules. No measurement of urinary iodine levels is another limitation of this study.

Another result of this study is that, patients with adenomatous polyps had 5 or more thyroid nodules compared to patients with hyperplastic polyps. This coexistence could not be explained easily. It can be speculated that APC gene mutation or others play a role in the development of adenomatous polyp and thyroid nodules [14].

As expected, thyroid nodules were more prevalent among patients aged 50 years or older, but, the prevalence of colonic polyp was not different between age groups. This could be explained by relatively narrow range of the age distribution (53.3 ± 11.4 years).

## 5. Conclusion

Thyroid nodules were detected more common in patients with colonic polyps. Further studies are needed to clarify this coexistence.
